# Supplementary material for: Spatial variation in coral reef fish and benthic communities in the central Saudi Arabian Red Sea
Source: PeerJ. 2017 Jun 6;5:e3410. doi: 10.7717/peerj.3410 (PMC5463981; doi:10.7717/peerj.3410)
Supplement: Table S1 — List of all fish species found on the 9 study reefs, the families or sub-families they belong to, and the trophic groups to which they were assigned, with corresponding a and b values obtained from FishBase (2014) and used in biomass calculations. All data were collected in May 2013 in the central Saudi Arabian Red Sea, with six 25 × 8 m belt transect replicates on each reef: 3 at 10 meters’ depth, and 3 at 2 meters’ depth. [file peerj-05-3410-s003.docx]

| Family/Sub-family | Species | Trophic group | a | b |
| --- | --- | --- | --- | --- |
| Acanthurinae | *Acanthurus gahhm* | Herbivore | 0.023 | 3.060 |
| Acanthurinae | *Acanthurus nigrofuscus* | Herbivore | 0.023 | 3.060 |
| Acanthurinae | *Acanthurus sohal* | Herbivore | 0.023 | 3.060 |
| Acanthurinae | *Ctenochaetus striatus* | Herbivore | 0.023 | 3.060 |
| Acanthurinae | *Zebrasoma desjardinii* | Herbivore | 0.034 | 2.861 |
| Acanthurinae | *Zebrasoma xanthurum* | Herbivore | 0.034 | 2.861 |
| Amphiprioninae | *Amphiprion bicinctus* | Herbivore | 0.020 | 3.000 |
| Anthiinae | *Pseudanthias squamipinnis* | Planktivore | 0.057 | 2.650 |
| Balistidae | *Balistapus undulatus* | Carnivore | 0.026 | 3.010 |
| Balistidae | *Pseudobalistes flavimarginatus* | Carnivore | 0.073 | 2.760 |
| Balistidae | *Sufflamen albicaudatus* | Carnivore | 0.030 | 2.957 |
| Belonidae | *Tylosurus choram* | Carnivore | 0.001 | 3.090 |
| Bodianinae | *Bodianus axillaris* | Carnivore | 0.011 | 3.039 |
| Bodianinae | *Bodianus diana* | Carnivore | 0.011 | 3.039 |
| Caesioninae | *Caesio lunaris* | Planktivore | 0.011 | 3.080 |
| Caesioninae | *Caesio striata* | Planktivore | 0.011 | 3.080 |
| Carangidae | *Atule mate* | Top Predator | 0.009 | 3.052 |
| Carangidae | *Carangoides bajad* | Top Predator | 0.009 | 3.052 |
| Carangidae | *Caranx melampygus* | Top Predator | 0.009 | 3.052 |
| Carcharhinidae | *Triaenodon obesus* | Top Predator | 0.009 | 3.052 |
| Chaetodontidae | *Chaetodon auriga* | Carnivore | 0.032 | 2.920 |
| Chaetodontidae | *Chaetodon austriacus* | Carnivore | 0.023 | 3.130 |
| Chaetodontidae | *Chaetodon fasciatus* | Carnivore | 0.023 | 3.130 |
| Chaetodontidae | *Chaetodon larvatus* | Carnivore | 0.026 | 3.100 |
| Chaetodontidae | *Chaetodon melannotus* | Carnivore | 0.027 | 3.049 |
| Chaetodontidae | *Chaetodon paucifasciatus* | Carnivore | 0.023 | 3.130 |
| Chaetodontidae | *Chaetodon semilarvatus* | Carnivore | 0.023 | 3.130 |
| Chaetodontidae | *Chaetodon trifascialis* | Carnivore | 0.035 | 2.860 |
| Chaetodontidae | *Heniochus intermedius* | Carnivore | 0.017 | 3.211 |
| Cheilininae | *Cheilinus abudjubbe* | Carnivore | 0.015 | 3.070 |
| Cheilininae | *Cheilinus quinquecinctus* | Carnivore | 0.015 | 3.000 |
| Cheilininae | *Epibulus insidiator* | Carnivore | 0.016 | 3.081 |
| Cirrhitidae | *Cirrhitus pinnulatus* | Carnivore | 0.021 | 3.000 |
| Cirrhitidae | *Paracirrhites forsteri* | Carnivore | 0.009 | 3.070 |
| Corinae | *Anampses twistii* | Carnivore | 0.020 | 3.000 |
| Corinae | *Coris cuvieri* | Carnivore | 0.003 | 3.489 |
| Corinae | *Halichoeres hortulanus* | Carnivore | 0.012 | 3.064 |
| Corinae | *Halichoeres scapularis* | Carnivore | 0.005 | 3.382 |
| Corinae | *Hemigymnus fasciatus* | Carnivore | 0.017 | 3.000 |
| Corinae | *Hologymnosus annulatus* | Carnivore | 0.004 | 3.010 |
| Corinae | *Stethojulis albavittata* | Carnivore | 0.013 | 3.077 |
| Corinae | *Thalassoma lunare* | Carnivore | 0.021 | 2.814 |
| Corinae | *Thalassoma purpureum* | Carnivore | 0.026 | 3.000 |
| Corinae | *Thalassoma rueppellii* | Carnivore | 0.021 | 2.814 |
| Dasyatidae | *Taeniura lymma* | Carnivore | 0.013 | 3.100 |
| Diodontidae | *Diodon hystrix* | Carnivore | 0.337 | 2.364 |
| Ephippidae | *Platax teira* | Herbivore | 0.043 | 2.975 |
| Epinephelinae | *Aethaloperca rogaa* | Carnivore | 0.030 | 3.000 |
| Epinephelinae | *Cephalopholis argus* | Carnivore | 0.012 | 3.120 |
| Epinephelinae | *Cephalopholis hemistiktos* | Carnivore | 0.022 | 3.000 |
| Epinephelinae | *Cephalopholis miniata* | Carnivore | 0.017 | 2.990 |
| Epinephelinae | *Epinephelus chlorostigma* | Carnivore | 0.011 | 3.078 |
| Epinephelinae | *Epinephelus stoliczkae* | Top Predator | 0.011 | 3.040 |
| Epinephelinae | *Epinephelus summana* | Top Predator | 0.021 | 3.000 |
| Epinephelinae | *Variola louti* | Carnivore | 0.014 | 3.117 |
| Fistulariidae | *Fistularia commersonii* | Carnivore | 0.001 | 3.000 |
| Holocentrinae | *Myripristis murdjan* | Planktivore | 0.019 | 3.034 |
| Holocentrinae | *Neoniphon sammara* | Carnivore | 0.021 | 3.036 |
| Holocentrinae | *Sargocentron caudimaculatum* | Carnivore | 0.019 | 3.050 |
| Holocentrinae | *Sargocentron diadema* | Carnivore | 0.012 | 3.120 |
| Holocentrinae | *Sargocentron spiniferum* | Carnivore | 0.019 | 3.050 |
| Kyphosinae | *Kyphosus sp.* | Herbivore | 0.023 | 3.055 |
| Labridae | *Gomphosus caeruleus* | Carnivore | 0.024 | 2.703 |
| Labridae | *Labroides dimidiatus* | Carnivore | 0.006 | 3.231 |
| Labridae | *Larabicus quadrilineatus* | Carnivore | 0.011 | 3.039 |
| Labridae | *Novaculichthys taeniourus* | Carnivore | 0.011 | 3.039 |
| Labridae | *Oxycheilinus digramma* | Carnivore | 0.049 | 2.450 |
| Labridae | *Oxycheilinus mentalis* | Carnivore | 0.049 | 2.450 |
| Labridae | *Paracheilinus octotaenia* | Carnivore | 0.011 | 3.039 |
| Lethrininae | *Lethrinus harak* | Carnivore | 0.017 | 3.037 |
| Lethrininae | *Lethrinus xanthochilus* | Carnivore | 0.022 | 2.940 |
| Lutjaninae | *Lutjanus bohar* | Top Predator | 0.016 | 3.059 |
| Lutjaninae | *Lutjanus ehrenbergii* | Top Predator | 0.003 | 3.335 |
| Lutjaninae | *Lutjanus fulviflamma* | Top Predator | 0.027 | 2.935 |
| Lutjaninae | *Lutjanus gibbus* | Top Predator | 0.023 | 3.060 |
| Lutjaninae | *Lutjanus kasmira* | Top Predator | 0.011 | 3.154 |
| Lutjaninae | *Lutjanus monostigma* | Top Predator | 0.022 | 2.913 |
| Monacanthidae | *Aluterus scriptus* | Carnivore | 0.823 | 1.814 |
| Monacanthidae | *Amanses scopas* | Carnivore | 0.022 | 2.922 |
| Monacanthidae | *Paraluteres arqat* | Carnivore | 0.010 | 3.150 |
| Monotaxinae | *Monotaxis grandoculis* | Carnivore | 0.027 | 2.960 |
| Mullidae | *Mulloidichthys vanicolensis* | Carnivore | 0.012 | 3.167 |
| Mullidae | *Parupeneus forsskali* | Carnivore | 0.010 | 3.110 |
| Muraeninae | *Gymnothorax javanicus* | Top Predator | 0.001 | 3.100 |
| Nasinae | *Naso brevirostris* | Herbivore | 0.060 | 2.743 |
| Nasinae | *Naso elegans* | Herbivore | 0.023 | 3.060 |
| Nasinae | *Naso hexacanthus* | Planktivore | 0.042 | 2.854 |
| Nasinae | *Naso unicornis* | Herbivore | 0.028 | 2.980 |
| Nemipteridae | *Scolopsis ghanam* | Carnivore | 0.012 | 2.990 |
| Ostraciidae | *Ostracion cubicus* | Carnivore | 0.115 | 2.550 |
| Pempheridae | *Pempheris sp.* | Carnivore | 0.012 | 3.064 |
| Pempheridae | *Pempheris vanicolensis* | Carnivore | 0.012 | 3.064 |
| Plectorhinchinae | *Plectorhinchus gaterinus* | Carnivore | 0.017 | 3.040 |
| Pomacanthidae | *Centropyge multispinis* | Carnivore | 0.031 | 2.885 |
| Pomacanthidae | *Pomacanthus asfur* | Carnivore | 0.034 | 2.968 |
| Pomacanthidae | *Pomacanthus imperator* | Carnivore | 0.034 | 2.968 |
| Pomacanthidae | *Pomacanthus maculosus* | Carnivore | 0.034 | 2.968 |
| Pomacanthidae | *Pygoplites diacanthus* | Herbivore | 0.031 | 2.885 |
| Pomacentrinae | *Abudefduf sexfasciatus* | Herbivore | 0.023 | 3.130 |
| Pomacentrinae | *Abudefduf vaigiensis* | Herbivore | 0.020 | 3.034 |
| Pomacentrinae | *Amblyglyphidodon flavilatus* | Planktivore | 0.023 | 3.130 |
| Pomacentrinae | *Amblyglyphidodon indicus* | Planktivore | 0.023 | 3.130 |
| Pomacentrinae | *Chromis dimidiata* | Planktivore | 0.057 | 2.650 |
| Pomacentrinae | *Chromis flavaxilla* | Planktivore | 0.057 | 2.650 |
| Pomacentrinae | *Chromis viridis* | Planktivore | 0.048 | 2.710 |
| Pomacentrinae | *Chromis weberi* | Planktivore | 0.057 | 2.650 |
| Pomacentrinae | *Dascyllus aruanus* | Planktivore | 0.050 | 2.736 |
| Pomacentrinae | *Dascyllus marginatus* | Planktivore | 0.018 | 3.000 |
| Pomacentrinae | *Dascyllus trimaculatus* | Planktivore | 0.060 | 2.850 |
| Pomacentrinae | *Neoglyphidodon melas* | Carnivore | 0.018 | 3.182 |
| Pomacentrinae | *Plectroglyphididon lacrymatus* | Herbivore | 0.061 | 2.635 |
| Pomacentrinae | *Pomacentrus sulfureus* | Herbivore | 0.030 | 2.870 |
| Pomacentrinae | *Pomacentrus trichrourus* | Herbivore | 0.031 | 3.000 |
| Pomacentrinae | *Stegastes nigricans* | Omnivore | 0.030 | 3.048 |
| Priacanthidae | *Priacanthus hamrur* | Carnivore | 0.031 | 2.788 |
| Pseudodacinae | *Pseudodax moluccanus* | Carnivore | 0.011 | 3.039 |
| Ptereleotridae | *Ptereleotris heteroptera* | Planktivore | 0.004 | 3.120 |
| Pteroinae | *Pterois miles* | Carnivore | 0.011 | 3.270 |
| Pteroinae | *Pterois radiata* | Carnivore | 0.011 | 3.270 |
| Scarinae | *Cetoscarus bicolor* | Herbivore | 0.020 | 3.000 |
| Scarinae | *Chlorurus gibbus* | Herbivore | 0.019 | 3.100 |
| Scarinae | *Chlorurus sordidus* | Herbivore | 0.019 | 3.100 |
| Scarinae | *Hipposcarus harid* | Herbivore | 0.013 | 3.050 |
| Scarinae | *Scarus ferrugineus* | Herbivore | 0.025 | 3.000 |
| Scarinae | *Scarus frenatus* | Herbivore | 0.025 | 3.000 |
| Scarinae | *Scarus niger* | Herbivore | 0.018 | 3.130 |
| Scarinae | *Scarus rivulatus* | Herbivore | 0.020 | 3.091 |
| Scarinae | *Scarus rubroviolaceus* | Herbivore | 0.014 | 3.109 |
| Serranidae | *Plectropomus areolatus* | Top Predator | 0.012 | 3.060 |
| Siganidae | *Siganus luridus* | Herbivore | 0.019 | 2.956 |
| Siganidae | *Siganus rivulatus* | Herbivore | 0.013 | 3.014 |
| Siganidae | *Siganus stellatus* | Herbivore | 0.014 | 3.138 |
| Sparidae | *Acanthopagrus bifasciatus* | Carnivore | 0.023 | 3.130 |
| Sparisomatinae | *Calotomus viridescens* | Herbivore | 0.012 | 3.167 |
| Tetraodontidae | *Arothron diadematus* | Carnivore | 0.017 | 2.960 |
| Total: | 136 species |  |  |  |
|  | 44 families |  |  |  |
