# Supplementary material for: Spatial variation in coral reef fish and benthic communities in the central Saudi Arabian Red Sea
Source: PeerJ. 2017 Jun 6;5:e3410. doi: 10.7717/peerj.3410 (PMC5463981; doi:10.7717/peerj.3410)
Supplement: Table S2 — A list of fish species that were observed during our surveys and that belong to 3 of the most commercially valued and heavily-targeted fishes by fishermen in Saudi Arabia (parrotfish, snappers, and groupers), and the families/subfamilies to which they belong. [file peerj-05-3410-s004.docx]

| **Group** | **Sub-family** | **Species** |
| --- | --- | --- |
| Parrotfishes | Scarinae | *Cetoscarus bicolor* |
|  | Scarinae | *Chlorurus gibbus* |
|  | Scarinae | *Chlorurus sordidus* |
|  | Scarinae | *Hipposcarus harid* |
|  | Scarinae | *Scarus ferrugineus* |
|  | Scarinae | *Scarus frenatus* |
|  | Scarinae | *Scarus niger* |
|  | Scarinae | *Scarus rivulatus* |
|  | Scarinae | *Scarus rubroviolaceus* |
|  | Sparisomatinae | *Calotomus viridescens* |
| Snappers | Lutjaninae | *Lutjanus bohar* |
|  | Lutjaninae | *Lutjanus ehrenbergii* |
|  | Lutjaninae | *Lutjanus fulviflamma* |
|  | Lutjaninae | *Lutjanus gibbus* |
|  | Lutjaninae | *Lutjanus kasmira* |
|  | Lutjaninae | *Lutjanus monostigma* |
| Groupers | Epinephelinae | *Aethaloperca rogaa* |
|  | Epinephelinae | *Cephalopholis argus* |
|  | Epinephelinae | *Cephalopholis hemistiktos* |
|  | Epinephelinae | *Cephalopholis miniata* |
|  | Epinephelinae | *Epinephelus chlorostigma* |
|  | Epinephelinae | *Epinephelus stoliczkae* |
|  | Epinephelinae | *Epinephelus summana* |
|  | Epinephelinae | *Variola louti* |
|  | Serraninae | *Plectropomus areolatus* |
| Parrotfish Total: | 10 species |  |
| Snappers Total: | 6 species |  |
| Groupers Total: | 9 species |  |
| Grand Total: | 25 species |  |
