# Supplementary material for: Spatial variation in coral reef fish and benthic communities in the central Saudi Arabian Red Sea
Source: PeerJ. 2017 Jun 6;5:e3410. doi: 10.7717/peerj.3410 (PMC5463981; doi:10.7717/peerj.3410)
Supplement: Table S3 — Mean and total fish biomass on the 9 study reefs in the central Saudi Arabian Red Sea expressed in mean kg/100 m2 (±SE). Each reef was surveyed using six replicate visual belt transects, three at 10 m and three at 2 m depth. “Habitat” indicates the location of each reef according to distance from shore, “Reef” is the name of each study reef (see main text for abbreviations). Values are divided by trophic group (planktivores, herbivores, carnivores, and top predators) and also shown as a Total for all groups combined. [file peerj-05-3410-s005.docx]

| habitat | reef | mean biomass kg/100m^2^ (±SE) | | | | | | | | | |
| --- | --- | --- | --- | --- | --- | --- | --- | --- | --- | --- | --- |
|  |  | planktivores | | herbivores | | carnivores | | top predators | | all fish | |
|  |  | 10 m | 2 m | 10 m | 2 m | 10 m | 2 m | 10 m | 2 m | 10 m | 2 m |
| offshore | RR | 1.07 (±0.5) | 1.94 (±0.1) | 7.12 (±5.3) | 11.01 (±3.4) | 3.32 (±0.4) | 1.04 (±0.2) | 2.21 (±0.9) | 1.75 (±0.9) | 13.71 (±5.9) | 15.74 (±14.6) |
|  | NR | 2.12 (±0.9) | 0.08 (±0.1) | 8.57 (±3.0) | 21.73 (±16.5) | 3.32 (±0.4) | 1.71 (±0.7) | 23.31 (±17.9) | 0.49 (±0.3) | 37.13 (±20.4) | 24.01 (±10.1) |
|  | AMR | 2.78 (±0.5) | 2.12 (±0.7) | 9.63 (±5.3) | 30.95 (±5.8) | 3.32 (±0.4) | 3.20 (±0.7) | 0.38 (±0.3) | 0.28 (±0.1) | 14.02 (±14.5) | 36.56 (±3.6) |
| midshore | FR | 0.67 (±0.1) | 0.50 (±0.2) | 1.87 (±0.7) | 7.64 (±3.4) | 3.32 (±0.4) | 2.50 (±0.7) | 2.06 (±1.5) | 0.59 (±0.6) | 7.26 (±5.6) | 11.22 (±1.4) |
|  | TWR | 0.96 (±0.8) | 0.20 (±0.2) | 2.46 (±0.9) | 8.24 (±1.0) | 3.32 (±0.4) | 2.80 (±1.3) | 0.23 (±0.2) | 0.61 (±0.5) | 5.93 (±3.6) | 11.85 (±5.6) |
|  | AHR | 1.78 (±0.6) | 0.84 (±0.2) | 2.61 (±0.8) | 21.52 (±2.3) | 3.32 (±0.4) | 2.40 (±0.7) | 2.92 (±2.8) | 0.61 (±0.4) | 11.54 (±2.2) | 25.37 (±1.1) |
| inshore | ASR | 0.49 (±0.1) | 0.09 (±0.1) | 2.93 (±18.1) | 22.65 (±18.1) | 3.32 (±0.4) | 4.07 (±1.5) | 0.00 (±0.1) | 0.20 (±0.1) | 4.68 (±3.3) | 27.01 (±18.6) |
|  | TR | 2.65 (±1.1) | 0.01 (±1.1) | 3.51 (±1.2) | 11.96 (±2.4) | 3.32 (±0.4) | 2.18 (±0.8) | 0.55 (±0.1) | 0.71 (±0.1) | 9.89 (±5.7) | 14.86 (±6.1) |
|  | EFR | 0.03 (±0.0) | 0.00 (±0.0) | 3.07 (±1.8) | 16.27 (±3.6) | 3.32 (±0.4) | 2.61 (±1.2) | 0.91 (±0.9) | 0.57 (±0.5) | 5.75 (±18.2) | 19.45 (±4) |
