# Supplementary material for: Spatial variation in coral reef fish and benthic communities in the central Saudi Arabian Red Sea
Source: PeerJ. 2017 Jun 6;5:e3410. doi: 10.7717/peerj.3410 (PMC5463981; doi:10.7717/peerj.3410)
Supplement: Table S4 — List of benthic categories recorded on the 9 study reefs. The taxonomic sub-categories (mostly genera) are listed in the second column as recorded during the surveys. A total of 25 scleractinian genera were recorded, at least 5 soft coral genera (the sub-category “Xeniidae” may have included more than one genus which were unidentifiable in the field), one zoanthid genus, and one genus of hydrozoan. Sponges and algae were recorded as general categories and the rest are non-living substrate categories. All data were collected using the line-intercept method in May 2013 in the central Saudi Arabian Red Sea on shorter (10 m long) subsets of the same transects used to collect fish data. There were 3 replicates at 10 m, and 3 at 2 m. [file peerj-05-3410-s006.docx]

| Benthic Category | Genus/Sub-category |
| --- | --- |
| Scleractinia | *Acropora* |
|  | *Astreopora* |
|  | *Ctenactis* |
|  | *Cyphastrea* |
|  | *Diploastrea* |
|  | *Echinopora* |
|  | *Echinophyllia* |
|  | *Dipsastraea* |
|  | *Favites* |
|  | *Fungia* |
|  | *Galaxea* |
|  | *Gardineroseris* |
|  | *Goniastrea* |
|  | *Goniopora* |
|  | *Gyrosmilia* |
|  | *Leptastrea* |
|  | *Lobophyllia* |
|  | *Montipora* |
|  | *Pavona* |
|  | *Platygyra* |
|  | *Pocillopora* |
|  | *Porites* |
|  | *Psammocora* |
|  | *Stylocoeniella* |
|  | *Stylophora* |
| Zoantharia | *Palythoa* |
| Octocorallia | *Rhytisma* |
|  | *Sarcophyton* |
|  | *Sinularia* |
|  | *Tubipora* |
|  | Xeniidae |
| Hydrozoans | *Millepora* |
| Sponge |  |
| Crustose coralline algae (CCA) |  |
| Turf algae |  |
| Other algae |  |
| Rock |  |
| Rubble |  |
| Sand |  |
