# Supplementary material for: Spatial variation in coral reef fish and benthic communities in the central Saudi Arabian Red Sea
Source: PeerJ. 2017 Jun 6;5:e3410. doi: 10.7717/peerj.3410 (PMC5463981; doi:10.7717/peerj.3410)
Supplement: Table S5 — Mean percent cover (±SE) of benthic categories recorded on the 9 study reefs in the central Saudi Arabian Red Sea. Reef names are shown as abbreviations in column headers and separated according to distance from shore. Data were collected on 10 m long transects at 10 m and 2 m depths using the line-intercept method. The category “hard corals” summarizes values for 25 scleractinian coral genera that were observed (listed in Table S4; “soft corals” summarize at least 6 genera; “hydrozoans” contained only the genus Millepora; “zoanthids” contained only the genus Palythoa; and the remaining categories were recorded as shown in the table. “CCA” stands for crustose coralline algae. [file peerj-05-3410-s007.docx]

| Benthic category | Mean percent cover (±SE) | | | | | | | | |
| --- | --- | --- | --- | --- | --- | --- | --- | --- | --- |
|  | offshore | | | midshore | | | inshore | | |
|  | RR | NR | AMR | FR | TWR | AHR | ASR | TR | EFR |
| Hard corals | 26.4 (±4.6) | 21.3 (±2.5) | 28.6 (±2.0) | 26.3 (±4.3) | 24.8 (±4.9) | 30.7 (±3.7) | 8.4 (±3.3) | 14.9 (±2.8) | 13.2 (±1.8) |
| Soft corals | 0.3 (±0.3) | 15.7 (±2.0) | 8.2 (±2.8) | 16.7 (±5.1) | 5.4 (±0.9) | 5.8 (±2.3) | 3 (±1.4) | 5.9 (±2.8) | 3.7 (±2.6) |
| Zoanthids | 0.0 (±0.0) | 0.0 (±0.0) | 0.0 (±0.0) | 0.0 (±0.0) | 0.0 (±0.0) | 0.0 (±0.0) | 0.0 (±0.0) | 1.7  (±0.9) | 0.0 (±0.0) |
| Hydrozoans | 1.5 (±1.2) | 0.3 (±0.2) | 1.8 (±1.2) | 1.7 (±1.7) | 0.0 (±0.0) | 0.6 (±0.3) | 0.0 (±0.0) | 0.2 (±0.2) | 0.0 (±0.0) |
| Sponges | 0.6 (±0.6) | 0.2 (±0.2) | 0.7 (±0.4) | 0.5 (±0.5) | 3.8 (±2.5) | 1.6 (±0.5) | 0.9 (±0.5) | 0.3 (±0.3) | 2.6 (±1.5) |
| CCA | 21.9 (±9.7) | 9.4 (±2.1) | 29.3 (±6.5) | 8.1 (±3.9) | 2.5 (±1.5) | 26.1 (±8.2) | 9.7 (±5.0) | 9.8 (±3.7) | 0.2 (±0.2) |
| Turf algae | 0.0 (±0.0) | 0.0 (±0.0) | 0.3 (±0.3) | 1.8 (±1.8) | 3.7 (±0.8) | 7.9 (±5.1) | 2.4 (±1.1) | 11.7 (±5.1) | 14.3 (±6.5) |
| Other algae | 0.0 (±0.0) | 0.0 (±0.0) | 0.2 (±0.2) | 0.1 (±0.1) | 0.3 (±0.3) | 0.4 (±0.4) | 0.0 (±0.0) | 0.2 (±0.2) | 0.0 (±0.0) |
| Rock | 35.5 (±4.0) | 52.8 (±2.3) | 30.5 (±7.0) | 31.3 (±6.0) | 36.6 (±5.7) | 26.9 (±7.6) | 40.9 (±7.6) | 43.4 (±3.6) | 50.2 (±5.3) |
| Rubble | 14 (±7.6) | 0.4 (±0.3) | 0.5 (±0.5) | 6.9 (±5.4) | 13.1 (±2.4) | 0.0 (±0.0) | 12.6 (±4.6) | 8.6 (±3.6) | 2.8 (±0.9) |
| Sand | 0.0 (±0.0) | 0.0 (±0.0) | 0.0 (±0.0) | 6.5 (±3.5) | 9.8 (±6.2) | 0.0 (±0.0) | 22.2 (±9.9) | 3.3 (±2.0) | 13 (±7.3) |
