# Supplementary material for: Spatial variation in coral reef fish and benthic communities in the central Saudi Arabian Red Sea
Source: PeerJ. 2017 Jun 6;5:e3410. doi: 10.7717/peerj.3410 (PMC5463981; doi:10.7717/peerj.3410)
Supplement: Table S6 — A list of all non-metric multidimensional scaling (NMDS), analyses of similarity (ANOSIM), and similarity percentage (SIMPER) analyses performed on the fish and benthic data collected on the 9 study reefs in the central Saudi Arabian Red Sea. The first column indicates which dataset was used (all data were log (x+1)-transformed); the second column indicates which subset of the dataset was included in the analysis (whether all replicates were used, replicates from a certain depth were used, and/or reef averages were used); the third column contains brief descriptions of NMDS plots produced; the fourth column shows the 2D stress for the plots described in the third column (>0.2 indicates poor representation of data in 2-dimensional space, <0.1 indicates excellent representation); the fifth column shows the global R statistic for ANOSIM (as the value approaches 1, the separation of groups analyzed is larger); the sixth column shows the significance of the separation indicated by the R statistic (values below 5% are considered significant); the seventh column shows the SIMPER percent average dissimilarity between the groups analyzed; and the last column lists some of the highest-contributing species/categories to the dissimilarity and the percent of their contribution between parentheses. Cells that are filled with dashes indicate that ANOSIM and SIMPER were not performed for the corresponding analyses indicated in the second column. [file peerj-05-3410-s008.docx]

| Data analyzed | Analysis | NMDS results/comments | 2D stress | ANOSIM | | SIMPER | |
| --- | --- | --- | --- | --- | --- | --- | --- |
|  |  |  |  | Global R statistic | Significance (%) | Average dissimilarity (%) | Top contributing species/categories (%) |
| Fish biomass (kg/transect) |  |  |  |  |  |  |  |
|  | All replicates, all depths | Poor but significant separation between shallow and deep replicates with more dissimilarity within groups than between them. | 0.27 | 0.31 | 0.1 | 85.2 | *Acanthurus sohal (11.6), Stegastes nigricans (5.8), Caesio lunaris (4.6)* |
|  | Reef averages, all depths | Poor separation between inshore and other reefs, with more dissimilarity within groups than between them. | 0.04 | 0.6 | 2.4 | 56 | *Stegastes nigricans (8.3), Pseudanthias squamipinnis (6.2), Thalassoma ruepellii (4.1)* |
|  | 10 m replicates only | Poor separation between 2 inshore reefs and all other reefs with more dissimilarity within groups than between them. | 0.24 | - | - | - | *-* |
|  | 10 m reef averages | Strong significant separation between 2 inshore reefs and all other reefs with high dissimilarity between the 2 inshore reefs. | 0.01 | 0.94 | 2.8 | 83.7 | *Caesio lunaris (7.9), Abudefduf sexfasciatus (5.0), Naso unicornis (4.6)* |
|  | 2 m replicates only | Poor separation between inshore reefs and all other reefs with more dissimilarity within groups than between them. | 0.21 | - | - | - | *-* |
|  | 2 m reef averages | Slight separation between inshore and other reefs with more dissimilarity within groups than between them. | 0.08 | 0.78 | 1.2 | 71 | *Stegastes nigricans (14.6), Acanthurus sohal (10.8), Naso unicornis (5.4)* |
| Fish density (fish/transect) |  |  |  |  |  |  |  |
|  | All replicates, all depths | Poor but significant separation between shallow and deep replicates with more dissimilarity within groups than between them. | 0.21 | 0.23 | 0.1 | 72.7 | *Chromis dimidiata (4.2), C. flavaxilla (3.9), Acanthurus sohal (3.5)* |
|  | Reef averages, all depths | Poor but significant separation between inshore and other reefs with more dissimilarity within groups than between them. | 0.07 | 0.75 | 1.2 | 56.7 | *Chromis dimidiata (9.5), Caesio lunaris (7.9), Chromis flavaxilla (6.7)* |
|  | 10 m replicates | Clear separation between 2 inshore reefs and all other reefs with more dissimilarity between the 2 inshore reefs than between groups. | 0.12 | - | - | - | *-* |
|  | 10 m reef averages | Clear and significant separation between 2 inshore reefs and all other reefs with more dissimilarity between the 2 inshore reefs than between groups. | 0.01 | 0.99 | 2.8 | 75.3 | *Chromis dimidiata + C. flavaxilla (14.4), Caesio lunaris + Pseudanthias squamipinnis (10.8)* |
|  | 2 m replicates | Poor separation between inshore reefs and all other reefs with more dissimilarity within groups than between them. | 0.19 | - | - | - | *-* |
|  | 2 m reef averages | Slight, yet significant, separation between inshore and other reefs with more dissimilarity within groups than between them. | 0.05 | 0.77 | 1.2 | 56.8 | *Stegastes nigricans (6.8), Chromis dimidiata + C. flavaxilla (8.5), Acanthurus nigrofuscus (3.8)* |
| Benthic cover (%) |  |  |  |  |  |  |  |
|  | All replicates, all depths | Poor but significant separation between shallow and deep replicates with more dissimilarity within groups than between them. | 0.17 | 0.45 | 0.1 | 57.4 | CCA *(14.0),* rubble + sand *(18.8),* turf algae *(7.4), Pocillopora (7.0)* |
|  | Reef averages, all depths | Poor separation between inshore reefs and all other reefs with more dissimilarity within groups than between them. | 0.08 | 0.65 | 1.2 | 41.3 | Sand *(13.1), Pocillopora (12.2),* CCA *(11.3),* turf algae *(10.2)* |
|  | 10 m replicates | No clear separation of groups. | 0.15 | - | - | - | *-* |
|  | 10 m reef averages | Separation of inshore plus 2 midshelf reefs from all other reefs, with more dissimilarity within than between groups. | 0.08 | 0.78 | 0.8 | 48.7 | Sand + rubble *(32), CCA (9.2),* Xeniidae *(5.1), Porites (5.1)* |
|  | 2 m replicates | Poor separation between inshore reefs and all other reefs with more dissimilarity within groups than between them. | 0.15 | - | - | - | *-* |
|  | 2 m reef averages | Clear separation between inshore reefs and all other reefs with more dissimilarity within groups than between them. | 0.08 | 0.82 | 1.2 | 50.8 | Turf algae *(14.6), Pocillopora (14.3),* CCA *(13.5),* rock *(10.4), Porites (10.0),* Xeniidae *(9.6)* |
| Fish density (fish/transect) and benthic cover (%) |  |  |  |  |  |  |  |
|  | Reef averages, all depths | Slightly better separation between inshore and other reefs compared to fish counts or benthic cover alone. | 0.04 | 0.74 | 1.2 | 49.8 | *Stegastes nigricans (4.9), Chromis dimidiata (4.6), Caesio lunaris (3.8)* |
